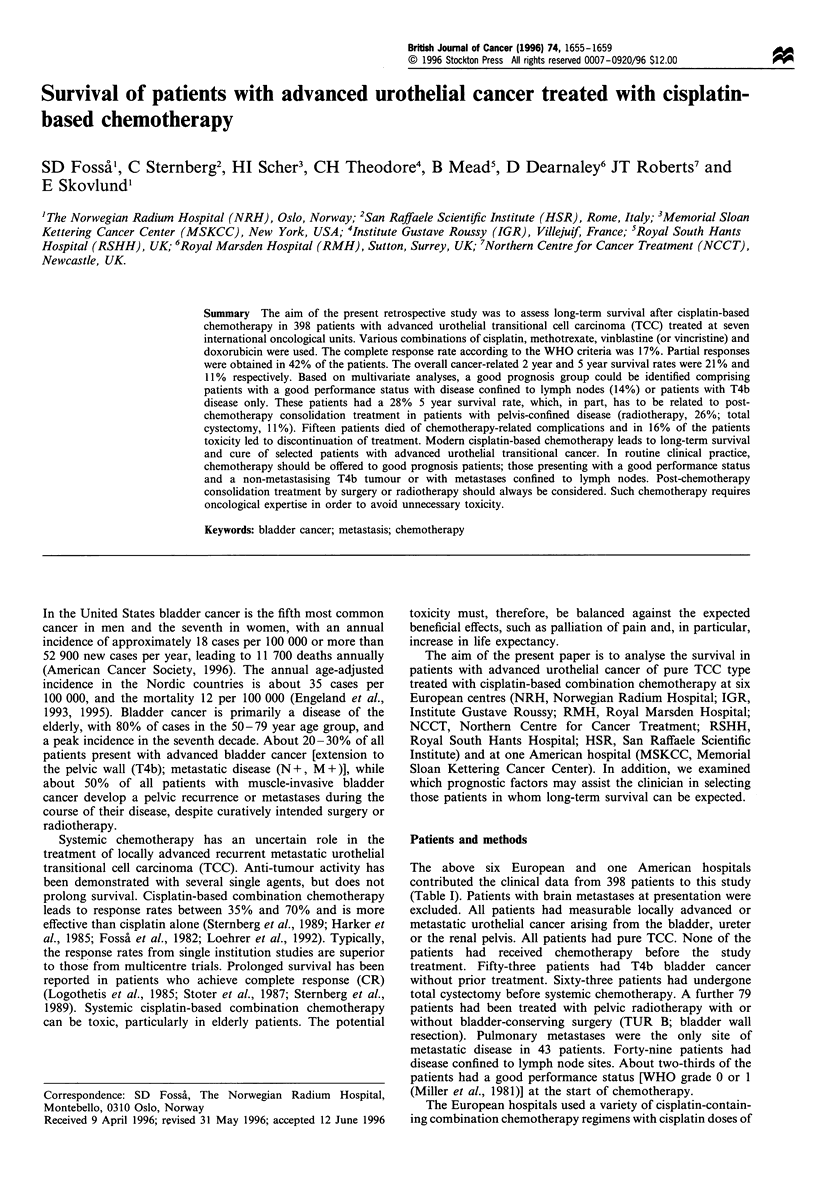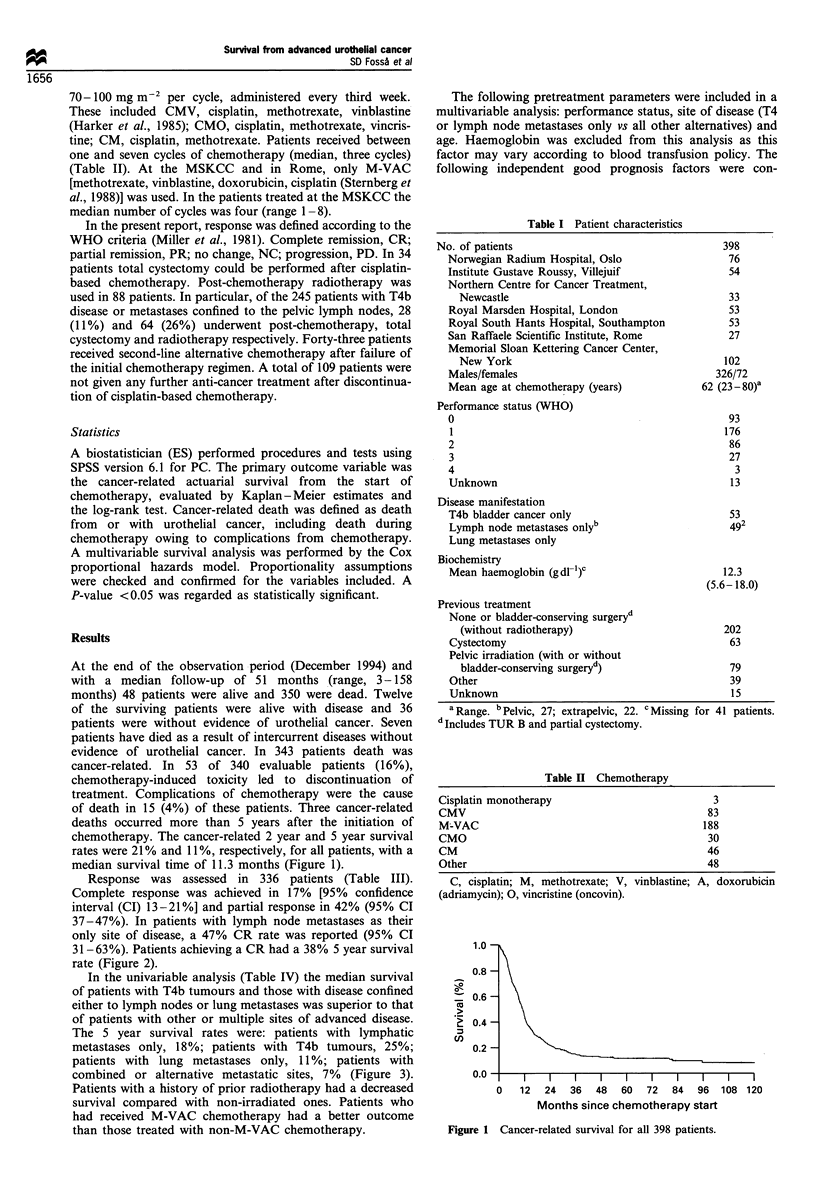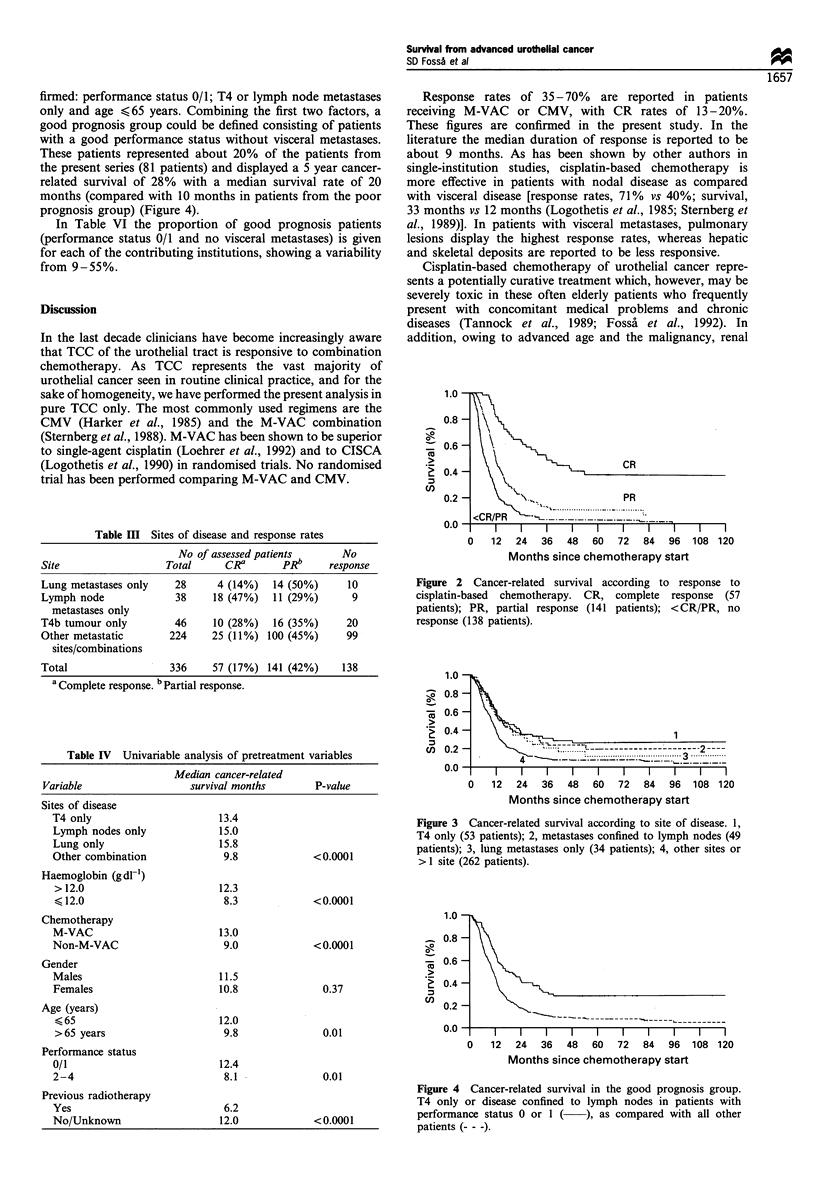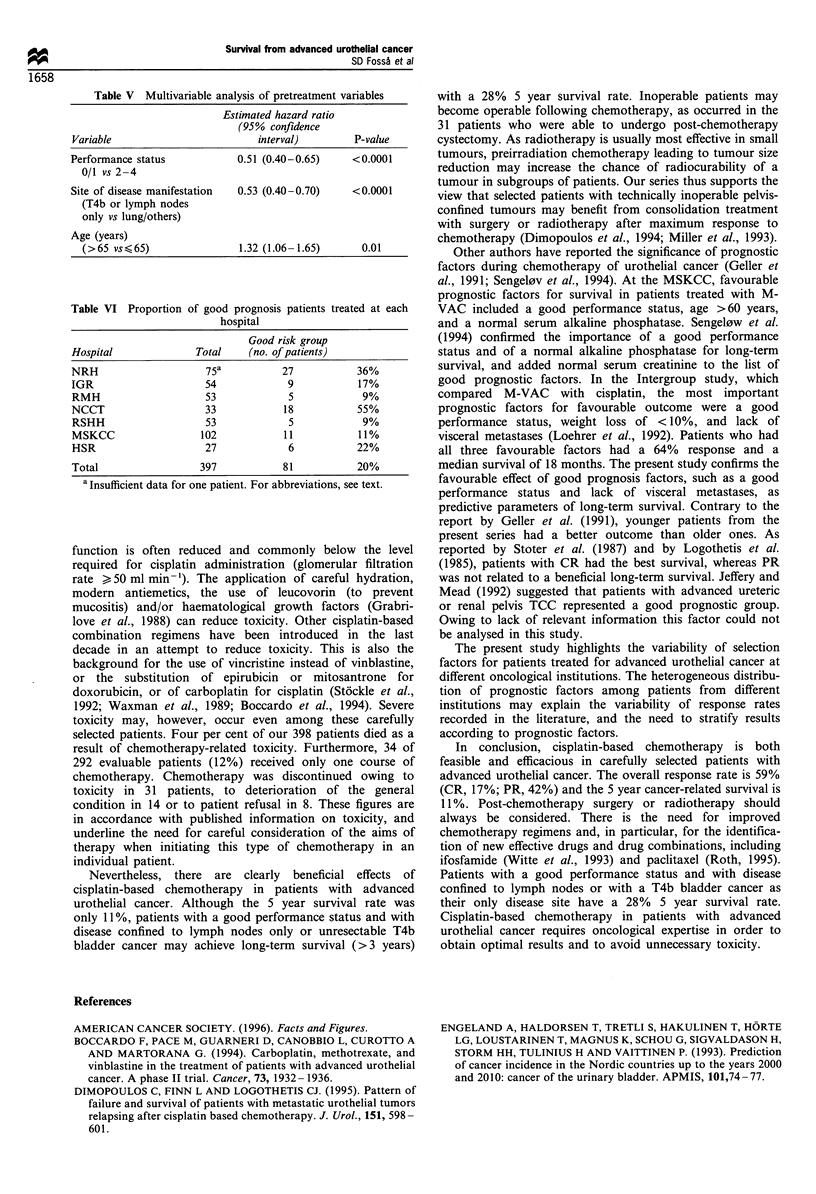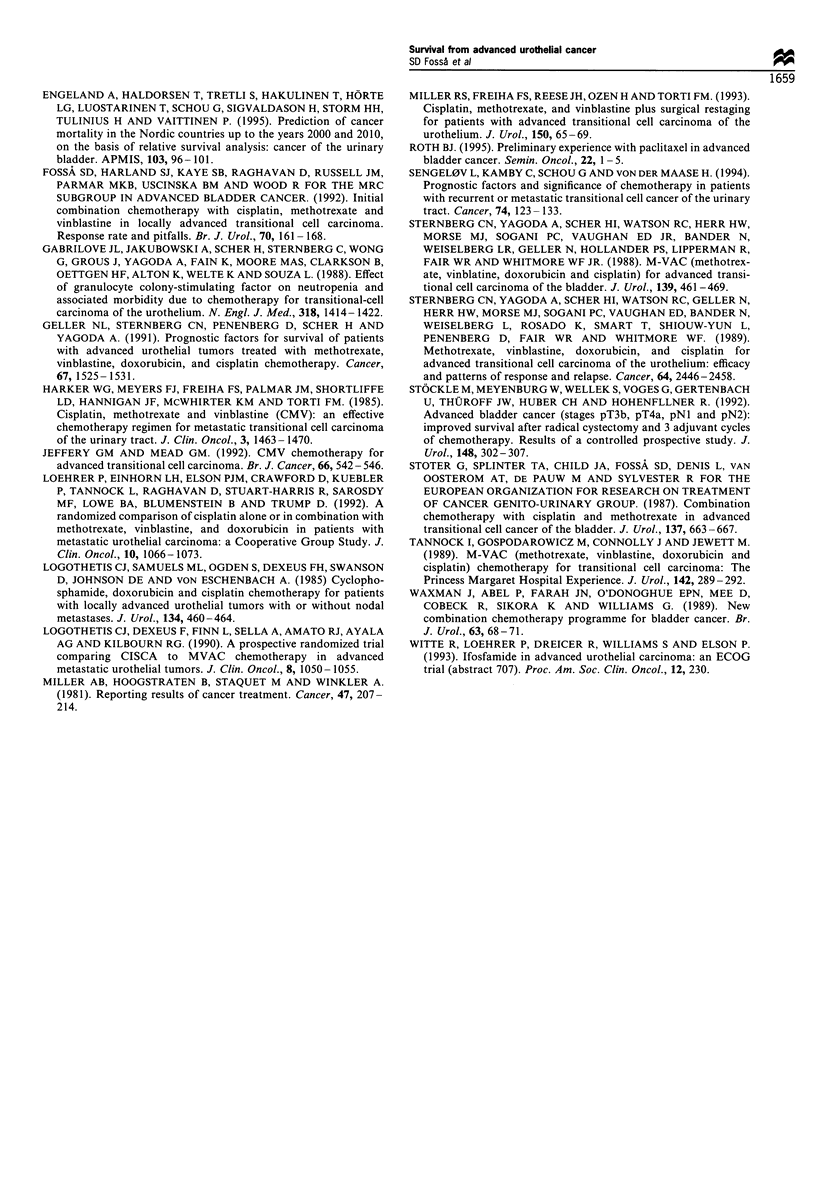# Survival of patients with advanced urothelial cancer treated with cisplatin-based chemotherapy.

**DOI:** 10.1038/bjc.1996.605

**Published:** 1996-11

**Authors:** S. D. Fosså, C. Sternberg, H. I. Scher, C. H. Theodore, B. Mead, D. Dearnaley, J. T. Roberts, E. Skovlund

**Affiliations:** The Norwegian Radium Hospital, Oslo.

## Abstract

The aim of the present retrospective study was to assess long-term survival after cisplatin-based chemotherapy in 398 patients with advanced urothelial transitional cell carcinoma (TCC) treated at seven international oncological units. Various combinations of cisplatin, methotrexate, vinblastine (or vincristine) and doxorubicin were used. The complete response rate according to the WHO criteria was 17%. Partial responses were obtained in 42% of the patients. The overall cancer-related 2 year and 5 year survival rates were 21% and 11% respectively. Based on multivariate analyses, a good prognosis group could be identified comprising patients with a good performance status with disease confined to lymph nodes (14%) or patients with T4b disease only. These patients had a 28% 5 year survival rate, which, in part, has to be related to post-chemotherapy consolidation treatment in patients with pelvis-confined disease (radiotherapy, 26%; total cystectomy, 11%). Fifteen patients died of chemotherapy-related complications and in 16% of the patients toxicity led to discontinuation of treatment. Modern cisplatin-based chemotherapy leads to long-term survival and cure of selected patients with advanced urothelial transitional cancer. In routine clinical practice, chemotherapy should be offered to good prognosis patients; those presenting with a good performance status and a non-metastasising T4b tumour or with metastases confined to lymph nodes. Post-chemotherapy consolidation treatment by surgery or radiotherapy should always be considered. Such chemotherapy requires oncological expertise in order to avoid unnecessary toxicity.


British Journal of Cancer (1996) 74, 1655-1659
© 1996 Stockton Press All rights reserved 0007-0920/96 $12.00

### Survival of patients with advanced urothelial cancer treated with cisplatin-based chemotherapy

SD Fosså[1], C Sternberg[2], HI Scher[3], CH Theodore[4], B Mead[5], D Dearnaley[6] JT Roberts[7] and E Skovlund[1]

[1]*The Norwegian Radium Hospital (NRH), Oslo, Norway;* [2]*San Raffaele Scientific Institute (HSR), Rome, Italy;* [3]*Memorial Sloan Kettering Cancer Center (MSKCC), New York, USA;* [4]*Institute Gustave Roussy (IGR), Villejuif, France;* [5]*Royal South Hants Hospital (RSHH), UK;* [6]*Royal Marsden Hospital (RMH), Sutton, Surrey, UK;* [7]*Northern Centre for Cancer Treatment (NCCT), Newcastle, UK.*

**Summary** The aim of the present retrospective study was to assess long-term survival after cisplatin-based chemotherapy in 398 patients with advanced urothelial transitional cell carcinoma (TCC) treated at seven international oncological units. Various combinations of cisplatin, methotrexate, vinblastine (or vincristine) and doxorubicin were used. The complete response rate according to the WHO criteria was 17%. Partial responses were obtained in 42% of the patients. The overall cancer-related 2 year and 5 year survival rates were 21% and 11% respectively. Based on multivariate analyses, a good prognosis group could be identified comprising patients with a good performance status with disease confined to lymph nodes (14%) or patients with T4b disease only. These patients had a 28% 5 year survival rate, which, in part, has to be related to post-chemotherapy consolidation treatment in patients with pelvis-confined disease (radiotherapy, 26%; total cystectomy, 11%). Fifteen patients died of chemotherapy-related complications and in 16% of the patients toxicity led to discontinuation of treatment. Modern cisplatin-based chemotherapy leads to long-term survival and cure of selected patients with advanced urothelial transitional cancer. In routine clinical practice, chemotherapy should be offered to good prognosis patients; those presenting with a good performance status and a non-metastasising T4b tumour or with metastases confined to lymph nodes. Post-chemotherapy consolidation treatment by surgery or radiotherapy should always be considered. Such chemotherapy requires oncological expertise in order to avoid unnecessary toxicity.

**Keywords:** bladder cancer; metastasis; chemotherapy

In the United States bladder cancer is the fifth most common cancer in men and the seventh in women, with an annual incidence of approximately 18 cases per 100 000 or more than 52 900 new cases per year, leading to 11 700 deaths annually (American Cancer Society, 1996). The annual age-adjusted incidence in the Nordic countries is about 35 cases per 100 000, and the mortality 12 per 100 000 (Engeland *et al.*, 1993, 1995). Bladder cancer is primarily a disease of the elderly, with 80% of cases in the 50–79 year age group, and a peak incidence in the seventh decade. About 20–30% of all patients present with advanced bladder cancer [extension to the pelvic wall (T4b); metastatic disease (N+, M+)], while about 50% of all patients with muscle-invasive bladder cancer develop a pelvic recurrence or metastases during the course of their disease, despite curatively intended surgery or radiotherapy.

Systemic chemotherapy has an uncertain role in the treatment of locally advanced recurrent metastatic urothelial transitional cell carcinoma (TCC). Anti-tumour activity has been demonstrated with several single agents, but does not prolong survival. Cisplatin-based combination chemotherapy leads to response rates between 35% and 70% and is more effective than cisplatin alone (Sternberg *et al.*, 1989; Harker *et al.*, 1985; Fosså *et al.*, 1982; Loehrer *et al.*, 1992). Typically, the response rates from single institution studies are superior to those from multicentre trials. Prolonged survival has been reported in patients who achieve complete response (CR) (Logothetis *et al.*, 1985; Stoter *et al.*, 1987; Sternberg *et al.*, 1989). Systemic cisplatin-based combination chemotherapy can be toxic, particularly in elderly patients. The potential toxicity must, therefore, be balanced against the expected beneficial effects, such as palliation of pain and, in particular, increase in life expectancy.

The aim of the present paper is to analyse the survival in patients with advanced urothelial cancer of pure TCC type treated with cisplatin-based combination chemotherapy at six European centres (NRH, Norwegian Radium Hospital; IGR, Institute Gustave Roussy; RMH, Royal Marsden Hospital; NCCT, Northern Centre for Cancer Treatment; RSHH, Royal South Hants Hospital; HSR, San Raffaele Scientific Institute) and at one American hospital (MSKCC, Memorial Sloan Kettering Cancer Center). In addition, we examined which prognostic factors may assist the clinician in selecting those patients in whom long-term survival can be expected.

### Patients and methods

The above six European and one American hospitals contributed the clinical data from 398 patients to this study (Table I). Patients with brain metastases at presentation were excluded. All patients had measurable locally advanced or metastatic urothelial cancer arising from the bladder, ureter or the renal pelvis. All patients had pure TCC. None of the patients had received chemotherapy before the study treatment. Fifty-three patients had T4b bladder cancer without prior treatment. Sixty-three patients had undergone total cystectomy before systemic chemotherapy. A further 79 patients had been treated with pelvic radiotherapy with or without bladder-conserving surgery (TUR B; bladder wall resection). Pulmonary metastases were the only site of metastatic disease in 43 patients. Forty-nine patients had disease confined to lymph node sites. About two-thirds of the patients had a good performance status [WHO grade 0 or 1 (Miller *et al.*, 1981)] at the start of chemotherapy.

The European hospitals used a variety of cisplatin-containing combination chemotherapy regimens with cisplatin doses of

Correspondence: SD Fosså, The Norwegian Radium Hospital, Montebello, 0310 Oslo, Norway
Received 9 April 1996; revised 31 May 1996; accepted 12 June 1996

70–100 mg m$^{-2}$ per cycle, administered every third week. These included CMV, cisplatin, methotrexate, vinblastine (Harker *et al.*, 1985); CMO, cisplatin, methotrexate, vincristine; CM, cisplatin, methotrexate. Patients received between one and seven cycles of chemotherapy (median, three cycles) (Table II). At the MSKCC and in Rome, only M-VAC [methotrexate, vinblastine, doxorubicin, cisplatin (Sternberg *et al.*, 1988)] was used. In the patients treated at the MSKCC the median number of cycles was four (range 1–8).

In the present report, response was defined according to the WHO criteria (Miller *et al.*, 1981). Complete remission, CR; partial remission, PR; no change, NC; progression, PD. In 34 patients total cystectomy could be performed after cisplatin-based chemotherapy. Post-chemotherapy radiotherapy was used in 88 patients. In particular, of the 245 patients with T4b disease or metastases confined to the pelvic lymph nodes, 28 (11%) and 64 (26%) underwent post-chemotherapy, total cystectomy and radiotherapy respectively. Forty-three patients received second-line alternative chemotherapy after failure of the initial chemotherapy regimen. A total of 109 patients were not given any further anti-cancer treatment after discontinuation of cisplatin-based chemotherapy.

### *Statistics*

A biostatistician (ES) performed procedures and tests using SPSS version 6.1 for PC. The primary outcome variable was the cancer-related actuarial survival from the start of chemotherapy, evaluated by Kaplan–Meier estimates and the log-rank test. Cancer-related death was defined as death from or with urothelial cancer, including death during chemotherapy owing to complications from chemotherapy. A multivariable survival analysis was performed by the Cox proportional hazards model. Proportionality assumptions were checked and confirmed for the variables included. A $P$-value $<0.05$ was regarded as statistically significant.

### Results

At the end of the observation period (December 1994) and with a median follow-up of 51 months (range, 3–158 months) 48 patients were alive and 350 were dead. Twelve of the surviving patients were alive with disease and 36 patients were without evidence of urothelial cancer. Seven patients have died as a result of intercurrent diseases without evidence of urothelial cancer. In 343 patients death was cancer-related. In 53 of 340 evaluable patients (16%), chemotherapy-induced toxicity led to discontinuation of treatment. Complications of chemotherapy were the cause of death in 15 (4%) of these patients. Three cancer-related deaths occurred more than 5 years after the initiation of chemotherapy. The cancer-related 2 year and 5 year survival rates were 21% and 11%, respectively, for all patients, with a median survival time of 11.3 months (Figure 1).

Response was assessed in 336 patients (Table III). Complete response was achieved in 17% [95% confidence interval (CI) 13–21%] and partial response in 42% (95% CI 37–47%). In patients with lymph node metastases as their only site of disease, a 47% CR rate was reported (95% CI 31–63%). Patients achieving a CR had a 38% 5 year survival rate (Figure 2).

In the univariable analysis (Table IV) the median survival of patients with T4b tumours and those with disease confined either to lymph nodes or lung metastases was superior to that of patients with other or multiple sites of advanced disease. The 5 year survival rates were: patients with lymphatic metastases only, 18%; patients with T4b tumours, 25%; patients with lung metastases only, 11%; patients with combined or alternative metastatic sites, 7% (Figure 3). Patients with a history of prior radiotherapy had a decreased survival compared with non-irradiated ones. Patients who had received M-VAC chemotherapy had a better outcome than those treated with non-M-VAC chemotherapy.

The following pretreatment parameters were included in a multivariable analysis: performance status, site of disease (T4 or lymph node metastases only *vs* all other alternatives) and age. Haemoglobin was excluded from this analysis as this factor may vary according to blood transfusion policy. The following independent good prognosis factors were con-

**Table I** Patient characteristics

| | |
|---|---|
| No. of patients | 398 |
| Norwegian Radium Hospital, Oslo | 76 |
| Institute Gustave Roussy, Villejuif | 54 |
| Northern Centre for Cancer Treatment, Newcastle | 33 |
| Royal Marsden Hospital, London | 53 |
| Royal South Hants Hospital, Southampton | 53 |
| San Raffaele Scientific Institute, Rome | 27 |
| Memorial Sloan Kettering Cancer Center, New York | 102 |
| Males/females | 326/72 |
| Mean age at chemotherapy (years) | 62 (23–80)[a] |
| Performance status (WHO) | |
| 0 | 93 |
| 1 | 176 |
| 2 | 86 |
| 3 | 27 |
| 4 | 3 |
| Unknown | 13 |
| Disease manifestation | |
| T4b bladder cancer only | 53 |
| Lymph node metastases only[b] | $49^{2}$ |
| Lung metastases only | |
| Biochemistry | |
| Mean haemoglobin (g dl$^{-1}$)[c] | 12.3 (5.6–18.0) |
| Previous treatment | |
| None or bladder-conserving surgery[d] (without radiotherapy) | 202 |
| Cystectomy | 63 |
| Pelvic irradiation (with or without bladder-conserving surgery[d]) | 79 |
| Other | 39 |
| Unknown | 15 |

[a] Range. [b] Pelvic, 27; extrapelvic, 22. [c] Missing for 41 patients. [d] Includes TUR B and partial cystectomy.

**Table II** Chemotherapy

| | |
|---|---|
| Cisplatin monotherapy | 3 |
| CMV | 83 |
| M-VAC | 188 |
| CMO | 30 |
| CM | 46 |
| Other | 48 |

C, cisplatin; M, methotrexate; V, vinblastine; A, doxorubicin (adriamycin); O, vincristine (oncovin).

**Figure 1** Cancer-related survival for all 398 patients.

firmed: performance status 0/1; T4 or lymph node metastases only and age ⩽65 years. Combining the first two factors, a good prognosis group could be defined consisting of patients with a good performance status without visceral metastases. These patients represented about 20% of the patients from the present series (81 patients) and displayed a 5 year cancer-related survival of 28% with a median survival rate of 20 months (compared with 10 months in patients from the poor prognosis group) (Figure 4).

In Table VI the proportion of good prognosis patients (performance status 0/1 and no visceral metastases) is given for each of the contributing institutions, showing a variability from 9–55%.

### Discussion

In the last decade clinicians have become increasingly aware that TCC of the urothelial tract is responsive to combination chemotherapy. As TCC represents the vast majority of urothelial cancer seen in routine clinical practice, and for the sake of homogeneity, we have performed the present analysis in pure TCC only. The most commonly used regimens are the CMV (Harker *et al.*, 1985) and the M-VAC combination (Sternberg *et al.*, 1988). M-VAC has been shown to be superior to single-agent cisplatin (Loehrer *et al.*, 1992) and to CISCA (Logothetis *et al.*, 1990) in randomised trials. No randomised trial has been performed comparing M-VAC and CMV.

**Table III** Sites of disease and response rates

| *Site* | *No of assessed patients* *Total* | *CR*[a] | *PR*[b] | *No response* |
|---|---|---|---|---|
| Lung metastases only | 28 | 4 (14%) | 14 (50%) | 10 |
| Lymph node metastases only | 38 | 18 (47%) | 11 (29%) | 9 |
| T4b tumour only | 46 | 10 (28%) | 16 (35%) | 20 |
| Other metastatic sites/combinations | 224 | 25 (11%) | 100 (45%) | 99 |
| Total | 336 | 57 (17%) | 141 (42%) | 138 |

[a] Complete response. [b] Partial response.

**Table IV** Univariable analysis of pretreatment variables

| *Variable* | *Median cancer-related survival months* | P-*value* |
|---|---|---|
| Sites of disease | | |
| T4 only | 13.4 | |
| Lymph nodes only | 15.0 | |
| Lung only | 15.8 | |
| Other combination | 9.8 | <0.0001 |
| Haemoglobin ($g\,dl^{-1}$) | | |
| >12.0 | 12.3 | |
| ⩽12.0 | 8.3 | <0.0001 |
| Chemotherapy | | |
| M-VAC | 13.0 | |
| Non-M-VAC | 9.0 | <0.0001 |
| Gender | | |
| Males | 11.5 | |
| Females | 10.8 | 0.37 |
| Age (years) | | |
| ⩽65 | 12.0 | |
| >65 years | 9.8 | 0.01 |
| Performance status | | |
| 0/1 | 12.4 | |
| 2–4 | 8.1 | 0.01 |
| Previous radiotherapy | | |
| Yes | 6.2 | |
| No/Unknown | 12.0 | <0.0001 |

Response rates of 35–70% are reported in patients receiving M-VAC or CMV, with CR rates of 13–20%. These figures are confirmed in the present study. In the literature the median duration of response is reported to be about 9 months. As has been shown by other authors in single-institution studies, cisplatin-based chemotherapy is more effective in patients with nodal disease as compared with visceral disease [response rates, 71% *vs* 40%; survival, 33 months *vs* 12 months (Logothetis *et al.*, 1985; Sternberg *et al.*, 1989)]. In patients with visceral metastases, pulmonary lesions display the highest response rates, whereas hepatic and skeletal deposits are reported to be less responsive.

Cisplatin-based chemotherapy of urothelial cancer represents a potentially curative treatment which, however, may be severely toxic in these often elderly patients who frequently present with concomitant medical problems and chronic diseases (Tannock *et al.*, 1989; Fosså *et al.*, 1992). In addition, owing to advanced age and the malignancy, renal

**Figure 2** Cancer-related survival according to response to cisplatin-based chemotherapy. CR, complete response (57 patients); PR, partial response (141 patients); <CR/PR, no response (138 patients).

**Figure 3** Cancer-related survival according to site of disease. 1, T4 only (53 patients); 2, metastases confined to lymph nodes (49 patients); 3, lung metastases only (34 patients); 4, other sites or >1 site (262 patients).

**Figure 4** Cancer-related survival in the good prognosis group. T4 only or disease confined to lymph nodes in patients with performance status 0 or 1 (——), as compared with all other patients (- - -).

**Table V** Multivariable analysis of pretreatment variables

| *Variable* | *Estimated hazard ratio (95% confidence interval)* | P-*value* |
|---|---|---|
| Performance status 0/1 *vs* 2–4 | 0.51 (0.40–0.65) | <0.0001 |
| Site of disease manifestation (T4b or lymph nodes only *vs* lung/others) | 0.53 (0.40–0.70) | <0.0001 |
| Age (years) (>65 *vs*≤65) | 1.32 (1.06–1.65) | 0.01 |

**Table VI** Proportion of good prognosis patients treated at each hospital

| *Hospital* | *Total* | *Good risk group (no. of patients)* | |
|---|---|---|---|
| NRH | 75[a] | 27 | 36% |
| IGR | 54 | 9 | 17% |
| RMH | 53 | 5 | 9% |
| NCCT | 33 | 18 | 55% |
| RSHH | 53 | 5 | 9% |
| MSKCC | 102 | 11 | 11% |
| HSR | 27 | 6 | 22% |
| Total | 397 | 81 | 20% |

[a] Insufficient data for one patient. For abbreviations, see text.

function is often reduced and commonly below the level required for cisplatin administration (glomerular filtration rate ≥50 ml $min^{-1}$). The application of careful hydration, modern antiemetics, the use of leucovorin (to prevent mucositis) and/or haematological growth factors (Grabrilove *et al.*, 1988) can reduce toxicity. Other cisplatin-based combination regimens have been introduced in the last decade in an attempt to reduce toxicity. This is also the background for the use of vincristine instead of vinblastine, or the substitution of epirubicin or mitosantrone for doxorubicin, or of carboplatin for cisplatin (Stöckle *et al.*, 1992; Waxman *et al.*, 1989; Boccardo *et al.*, 1994). Severe toxicity may, however, occur even among these carefully selected patients. Four per cent of our 398 patients died as a result of chemotherapy-related toxicity. Furthermore, 34 of 292 evaluable patients (12%) received only one course of chemotherapy. Chemotherapy was discontinued owing to toxicity in 31 patients, to deterioration of the general condition in 14 or to patient refusal in 8. These figures are in accordance with published information on toxicity, and underline the need for careful consideration of the aims of therapy when initiating this type of chemotherapy in an individual patient.

Nevertheless, there are clearly beneficial effects of cisplatin-based chemotherapy in patients with advanced urothelial cancer. Although the 5 year survival rate was only 11%, patients with a good performance status and with disease confined to lymph nodes only or unresectable T4b bladder cancer may achieve long-term survival (>3 years) with a 28% 5 year survival rate. Inoperable patients may become operable following chemotherapy, as occurred in the 31 patients who were able to undergo post-chemotherapy cystectomy. As radiotherapy is usually most effective in small tumours, preirradiation chemotherapy leading to tumour size reduction may increase the chance of radiocurability of a tumour in subgroups of patients. Our series thus supports the view that selected patients with technically inoperable pelvis-confined tumours may benefit from consolidation treatment with surgery or radiotherapy after maximum response to chemotherapy (Dimopoulos *et al.*, 1994; Miller *et al.*, 1993).

Other authors have reported the significance of prognostic factors during chemotherapy of urothelial cancer (Geller *et al.*, 1991; Sengeløv *et al.*, 1994). At the MSKCC, favourable prognostic factors for survival in patients treated with M-VAC included a good performance status, age >60 years, and a normal serum alkaline phosphatase. Sengeløw *et al.* (1994) confirmed the importance of a good performance status and of a normal alkaline phosphatase for long-term survival, and added normal serum creatinine to the list of good prognostic factors. In the Intergroup study, which compared M-VAC with cisplatin, the most important prognostic factors for favourable outcome were a good performance status, weight loss of <10%, and lack of visceral metastases (Loehrer *et al.*, 1992). Patients who had all three favourable factors had a 64% response and a median survival of 18 months. The present study confirms the favourable effect of good prognosis factors, such as a good performance status and lack of visceral metastases, as predictive parameters of long-term survival. Contrary to the report by Geller *et al.* (1991), younger patients from the present series had a better outcome than older ones. As reported by Stoter *et al.* (1987) and by Logothetis *et al.* (1985), patients with CR had the best survival, whereas PR was not related to a beneficial long-term survival. Jeffery and Mead (1992) suggested that patients with advanced ureteric or renal pelvis TCC represented a good prognostic group. Owing to lack of relevant information this factor could not be analysed in this study.

The present study highlights the variability of selection factors for patients treated for advanced urothelial cancer at different oncological institutions. The heterogeneous distribution of prognostic factors among patients from different institutions may explain the variability of response rates recorded in the literature, and the need to stratify results according to prognostic factors.

In conclusion, cisplatin-based chemotherapy is both feasible and efficacious in carefully selected patients with advanced urothelial cancer. The overall response rate is 59% (CR, 17%; PR, 42%) and the 5 year cancer-related survival is 11%. Post-chemotherapy surgery or radiotherapy should always be considered. There is the need for improved chemotherapy regimens and, in particular, for the identification of new effective drugs and drug combinations, including ifosfamide (Witte *et al.*, 1993) and paclitaxel (Roth, 1995). Patients with a good performance status and with disease confined to lymph nodes or with a T4b bladder cancer as their only disease site have a 28% 5 year survival rate. Cisplatin-based chemotherapy in patients with advanced urothelial cancer requires oncological expertise in order to obtain optimal results and to avoid unnecessary toxicity.

### References

AMERICAN CANCER SOCIETY. (1996). *Facts and Figures.*

BOCCARDO F, PACE M, GUARNERI D, CANOBBIO L, CUROTTO A AND MARTORANA G. (1994). Carboplatin, methotrexate, and vinblastine in the treatment of patients with advanced urothelial cancer. A phase II trial. *Cancer*, **73**, 1932–1936.

DIMOPOULOS C, FINN L AND LOGOTHETIS CJ. (1995). Pattern of failure and survival of patients with metastatic urothelial tumors relapsing after cisplatin based chemotherapy. *J. Urol.*, **151**, 598–601.

ENGELAND A, HALDORSEN T, TRETLI S, HAKULINEN T, HÖRTE LG, LOUSTARINEN T, MAGNUS K, SCHOU G, SIGVALDASON H, STORM HH, TULINIUS H AND VAITTINEN P. (1993). Prediction of cancer incidence in the Nordic countries up to the years 2000 and 2010: cancer of the urinary bladder. APMIS, **101**,74–77.

ENGELAND A, HALDORSEN T, TRETLI S, HAKULINEN T, HÖRTE LG, LUOSTARINEN T, SCHOU G, SIGVALDASON H, STORM HH, TULINIUS H AND VAITTINEN P. (1995). Prediction of cancer mortality in the Nordic countries up to the years 2000 and 2010, on the basis of relative survival analysis: cancer of the urinary bladder. APMIS, **103**, 96–101.

FOSSÅ SD, HARLAND SJ, KAYE SB, RAGHAVAN D, RUSSELL JM, PARMAR MKB, USCINSKA BM AND WOOD R FOR THE MRC SUBGROUP IN ADVANCED BLADDER CANCER. (1992). Initial combination chemotherapy with cisplatin, methotrexate and vinblastine in locally advanced transitional cell carcinoma. Response rate and pitfalls. *Br. J. Urol.*, **70**, 161–168.

GABRILOVE JL, JAKUBOWSKI A, SCHER H, STERNBERG C, WONG G, GROUS J, YAGODA A, FAIN K, MOORE MAS, CLARKSON B, OETTGEN HF, ALTON K, WELTE K AND SOUZA L. (1988). Effect of granulocyte colony-stimulating factor on neutropenia and associated morbidity due to chemotherapy for transitional-cell carcinoma of the urothelium. *N. Engl. J. Med.*, **318**, 1414–1422.

GELLER NL, STERNBERG CN, PENENBERG D, SCHER H AND YAGODA A. (1991). Prognostic factors for survival of patients with advanced urothelial tumors treated with methotrexate, vinblastine, doxorubicin, and cisplatin chemotherapy. *Cancer*, **67**, 1525–1531.

HARKER WG, MEYERS FJ, FREIHA FS, PALMAR JM, SHORTLIFFE LD, HANNIGAN JF, McWHIRTER KM AND TORTI FM. (1985). Cisplatin, methotrexate and vinblastine (CMV): an effective chemotherapy regimen for metastatic transitional cell carcinoma of the urinary tract. *J. Clin. Oncol.*, **3**, 1463–1470.

JEFFERY GM AND MEAD GM. (1992). CMV chemotherapy for advanced transitional cell carcinoma. *Br. J. Cancer*, **66**, 542–546.

LOEHRER P, EINHORN LH, ELSON PJM, CRAWFORD D, KUEBLER P, TANNOCK L, RAGHAVAN D, STUART-HARRIS R, SAROSDY MF, LOWE BA, BLUMENSTEIN B AND TRUMP D. (1992). A randomized comparison of cisplatin alone or in combination with methotrexate, vinblastine, and doxorubicin in patients with metastatic urothelial carcinoma: a Cooperative Group Study. *J. Clin. Oncol.*, **10**, 1066–1073.

LOGOTHETIS CJ, SAMUELS ML, OGDEN S, DEXEUS FH, SWANSON D, JOHNSON DE AND VON ESCHENBACH A. (1985) Cyclophosphamide, doxorubicin and cisplatin chemotherapy for patients with locally advanced urothelial tumors with or without nodal metastases. *J. Urol.*, **134**, 460–464.

LOGOTHETIS CJ, DEXEUS F, FINN L, SELLA A, AMATO RJ, AYALA AG AND KILBOURN RG. (1990). A prospective randomized trial comparing CISCA to MVAC chemotherapy in advanced metastatic urothelial tumors. *J. Clin. Oncol.*, **8**, 1050–1055.

MILLER AB, HOOGSTRATEN B, STAQUET M AND WINKLER A. (1981). Reporting results of cancer treatment. *Cancer*, **47**, 207–214.

MILLER RS, FREIHA FS, REESE JH, OZEN H AND TORTI FM. (1993). Cisplatin, methotrexate, and vinblastine plus surgical restaging for patients with advanced transitional cell carcinoma of the urothelium. *J. Urol.*, **150**, 65–69.

ROTH BJ. (1995). Preliminary experience with paclitaxel in advanced bladder cancer. *Semin. Oncol.*, **22**, 1–5.

SENGELØV L, KAMBY C, SCHOU G AND VON DER MAASE H. (1994). Prognostic factors and significance of chemotherapy in patients with recurrent or metastatic transitional cell cancer of the urinary tract. *Cancer*, **74**, 123–133.

STERNBERG CN, YAGODA A, SCHER HI, WATSON RC, HERR HW, MORSE MJ, SOGANI PC, VAUGHAN ED JR, BANDER N, WEISELBERG LR, GELLER N, HOLLANDER PS, LIPPERMAN R, FAIR WR AND WHITMORE WF JR. (1988). M-VAC (methotrexate, vinblatine, doxorubicin and cisplatin) for advanced transitional cell carcinoma of the bladder. *J. Urol.*, **139**, 461–469.

STERNBERG CN, YAGODA A, SCHER HI, WATSON RC, GELLER N, HERR HW, MORSE MJ, SOGANI PC, VAUGHAN ED, BANDER N, WEISELBERG L, ROSADO K, SMART T, SHIOUW-YUN L, PENENBERG D, FAIR WR AND WHITMORE WF. (1989). Methotrexate, vinblastine, doxorubicin, and cisplatin for advanced transitional cell carcinoma of the urothelium: efficacy and patterns of response and relapse. *Cancer*, **64**, 2446–2458.

STÖCKLE M, MEYENBURG W, WELLEK S, VOGES G, GERTENBACH U, THÜROFF JW, HUBER CH AND HOHENFLLNER R. (1992). Advanced bladder cancer (stages pT3b, pT4a, pN1 and pN2): improved survival after radical cystectomy and 3 adjuvant cycles of chemotherapy. Results of a controlled prospective study. *J. Urol.*, **148**, 302–307.

STOTER G, SPLINTER TA, CHILD JA, FOSSÅ SD, DENIS L, VAN OOSTEROM AT, DE PAUW M AND SYLVESTER R FOR THE EUROPEAN ORGANIZATION FOR RESEARCH ON TREATMENT OF CANCER GENITO-URINARY GROUP. (1987). Combination chemotherapy with cisplatin and methotrexate in advanced transitional cell cancer of the bladder. *J. Urol.*, **137**, 663–667.

TANNOCK I, GOSPODAROWICZ M, CONNOLLY J AND JEWETT M. (1989). M-VAC (methotrexate, vinblastine, doxorubicin and cisplatin) chemotherapy for transitional cell carcinoma: The Princess Margaret Hospital Experience. *J. Urol.*, **142**, 289–292.

WAXMAN J, ABEL P, FARAH JN, O'DONOGHUE EPN, MEE D, COBECK R, SIKORA K AND WILLIAMS G. (1989). New combination chemotherapy programme for bladder cancer. *Br. J. Urol.*, **63**, 68–71.

WITTE R, LOEHRER P, DREICER R, WILLIAMS S AND ELSON P. (1993). Ifosfamide in advanced urothelial carcinoma: an ECOG trial (abstract 707). *Proc. Am. Soc. Clin. Oncol.*, **12**, 230.